# A Further Look at Reading the Mind in the Eyes-Child Version: Association With Fluid Intelligence, Receptive Language, and Intergenerational Transmission in Typically Developing School-Aged Children

**DOI:** 10.3389/fpsyg.2020.586065

**Published:** 2020-12-07

**Authors:** Anna Maria Rosso, Arianna Riolfo

**Affiliations:** Department of Education, University of Genoa, Genoa, Italy

**Keywords:** reading the mind in the eyes, receptive language, fluid intelligence, theory-of-mind, intergenerational transmission

## Abstract

A number of tasks have been developed to measure the affective theory of mind (ToM), nevertheless, recent studies found that different affective ToM tasks do not correlate with each other, suggesting that further studies on affective ToM and its measurement are needed. More in-depth knowledge of the tools that are available to assess affective ToM is needed to decide which should be used in research and in clinical practice, and how to interpret results. The current study focuses on the Reading the Mind in the Eyes Test (RMET) primarily to investigate in a sample of 112 children the currently unexplored relationships in middle childhood between performance on the RMET and fluid intelligence. Relationships with receptive vocabulary, age, and sex were also investigated. Moreover, because studying the family's influence on children mentalization could have important implications in developing prevention and treatment interventions, this study offers a novel contribution to the field by exploring the family's influence on children's RMET performance. Although significant positive correlations were found among RMET-C performance, fluid intelligence, and receptive language, regression analysis revealed that fluid intelligence was the only predictor. No family influence was found on children's RMET performance. On the whole, results from the current study offer some support to the hypothesis that RMET-C is not a “pure” ToM task, specifically the effect of fluid intelligence on RMET performance should be taken into account when RMET is used both in research and in the clinical setting.

## Introduction

Mentalization refers, in a broad sense, to the human ability to interpret one's own and others' behavior in terms of intentional mental states (e.g., desires, needs, feelings, and beliefs) (Allen, 2003; Fonagy and Target, 2005).

Over the last decades several tasks have been designed to evaluate mentalization, and an extensive body of studies has focused on its development in non-clinical samples and its impairment in clinical groups. As Luyten et al. (2019) stated, mentalizing has become over time an umbrella concept that overlaps with Theory of Mind (ToM), so that in literature mentalization and ToM are often used as interchangeable terms. At first, developmental studies focused largely on children's understanding of false belief (Wimmer and Perner, 1983), then research moved toward the investigation of emotion decoding (Baron-Cohen et al., 2001a), pragmatic language comprehension (Happé, 1994), and mental state talk (Bartsch and Wellman, 1995), while more recently studies have focused on children's reflective functioning in the context of close attachment relationships (Ensink et al., 2015).

A vast array of studies support the hypothesis that the construct of mentalization is a complex and multifaceted one (for a review, Fonagy and Bateman, 2019) that includes distinct components underpinned by different neural correlates (Schurz et al., 2014; for a review).

A number of instruments have been designed to assess mentalization in adults and in children, however, to date, it is not really clear which component(s) of mentalization each tool actually measures. Warnell and Redcay (2019) administered a diverse set of ToM tasks to three different sample groups, each of which contained children of the same age, and found that at any age, receiving high scores on one task did not predict performance on another task designed to assess the same underlying ability. In middle childhood, this study did not find any significant correlation between the scores obtained on the children's version of the Reading the Mind in the Eyes (RMET-C; Baron-Cohen et al., 2001b), the Strange Stories (Happé, 1994), and the Faux Pas Task (Baron-Cohen et al., 1999). In addition, in middle childhood full-scale IQ was significantly related only to Strange Stories performance, suggesting that the association between ToM and intelligence should be investigated regarding each ToM component, rather than assumed regarding ToM as a unitary construct.

Some recent developmental studies on samples of preschool children (e.g., Lecce et al., 2015; Longobardi et al., 2017) offered support for a distinction between cognitive and affective ToM—namely the ability to attribute beliefs and/or intentions above and beyond the appearance vs. the ability to recognize and infer emotions and feelings.

In addition, Gallant et al. (2020) found that different affective ToM tasks did not correlate with each other in a sample of preschool children, supporting the hypothesis that diverse instruments measure distinct facets of affective ToM, consequently they suggested that further studies on affective ToM and its measurement are needed.

The current study aims primarily to investigate in a sample of school-aged children the **currently** unexplored relationship between performance on the RMET—a widely used affective ToM task—and fluid intelligence. Relationships among RMET performance, receptive vocabulary, age, and sex were also investigated.

Baron-Cohen et al. (1997, 2001a) developed the adult version of the RMET both to measure sensitive to subtle dysfunction in the domain of social cognition in adults with a diagnosis of autism or Asperger syndrome, and for use with adults of normal intelligence. RMET consists of 36 photographs of the eye region of the face of different actors and actresses. At the four corners of each photo there are four words (the target word and its three foils), the subject is simply required to choose the correct term.

The test was conceived as a measure of the individuals' ability to put themselves into the mind of the other person by tuning into their mental state. Consequently, Baron-Cohen et al. (2001a) defined the test as an advanced theory of mind task which requires having a mental state language and, at a quick and automatic level, matching the eyes in each photo to eye region expressions stored in one's memory as seen in the context of a particular mental state, and to choose the word the eyes in the photo most closely match. Baron-Cohen et al. (2001a) specified that RMET only implies the first stage of theory of mind, namely the attribution of the relevant mental state, whereas it does not include the second stage consisting of inferring the content of that mental state (e.g., in the case sadness was identified as the mental state, participants were not required to infer the reason why). The test includes a control task consisting of showing the same photographs again and asking the participant to determine the gender of the person based on his/her eyes. This control task, named Gender Recognition task, implies a non-mentalistic social cognition from the eyes as well as attention to the stimuli.

The final version of the RMET was able to detect meaningful individual differences with normal performance significantly below ceiling. In the original study no effect of general intelligence was observed in the non-clinical sample with regard to RMET performance, while a trend toward a female advantage was found. On the contrary, the most recent meta-analyses (Baker et al., 2014; Peñuelas-Calvo et al., 2019) found that intelligence played a significant role in adults' non-clinical performance on the RMET and that verbal and performance abilities equally contribute to this relationship. The better performance by females on RMET was confirmed by another meta-analysis (Kirkland et al., 2013). Recently, Baron-Cohen et al. (2015) too, in an extensive study, found that females outperformed males in non-clinical samples, while no sex difference was found in individuals with autism.

A child version (RMET-C; Baron-Cohen et al., 2001b), conceptually derived from the adult version, consists of 28 photographs of the eye region of female and male adult actors. Like the adult version, each illustration is accompanied by four words that refer to mental states (e.g. “hate,” “surprise,” “cross,” kind”). The child is asked to point to the one that best represents what that person feels or thinks. The Gender Recognition task may be used as a control test, like in the adult version.

Although the test has been very widely used, especially in the adult version, data regarding psychometric properties are rarely reported and are controversial, especially with regard to internal consistency that was found to be low in four studies (Voracek and Dressler, 2006; Harkness et al., 2010; Müller and Gmünder, 2014; Hayward and Homer, 2017), and minimally acceptable or acceptable in five other studies (Serafin and Surian, 2004; Dehning et al., 2012; Vellante et al., 2013; Girli, 2014; Vogindroukas et al., 2014). To our knowledge, only four studies have investigated internal coherence in the children's version (Girli, 2014; Müller and Gmünder, 2014; Vogindroukas et al., 2014; Hayward and Homer, 2017), and no studies have ever been conducted on an Italian sample. Test-retest stability was found to be acceptable for both the adult (Vellante et al., 2013) and the child version (Hallerbäck et al., 2009). Some studies confirmed the single factor structure assumed by Baron-Cohen et al. (2001a) both in the adult (Vellante et al., 2013) and in the child version (Carey and Cassels, 2013), while Olderbak et al. (2015) did not find a single-factor solution in the adult version.

RMET-C does not require reasoning about mental states but only recognizing them, thus it may be primarily considered a measure of affective mentalizing, mainly focused on emotion recognition (Oakley et al., 2016). Most studies (Rutherford et al., 2012; Ha et al., 2013; Fossati et al., 2014; Gallant et al., 2020) described RMET-C as a measure of affective ToM assessing the ability to understand the feeling of mental states. In addition, some studies (Lawrence et al., 2004; Carroll and Yung, 2006) found that it correlated with measures of empathy.

Although the child version of the RMET was used in several studies to compare ToM abilities in clinical and non-clinical samples, to our knowledge, no study has specifically aimed to investigate its relationship with verbal ability and non-verbal intelligence, nor with sex, age or family background.

Some studies reported data concerning the relationship between RMET-C performance and intelligence in typically developing school-aged children. Furthermore, these studies used different intelligence measures, making it hard to compare results. Some studies did not report a significant effect of intelligence on RMET-C performance (Sharp, 2008; Mary et al., 2016; Stevens et al., 2017), while Baribeau et al. (2015) found a significant effect, and Warnell and Redcay (2019) reported a trend toward statistical significance. Only two studies (Ibanez et al., 2013; Levy and Milgram, 2016) investigated the relationship between fluid intelligence and RMET-C performance, and reported a significant association.

Regarding sex, in children samples findings were contradictory in the few studies reporting data. A small effect of sex on RMET-C performance was found in two studies (Chapman et al., 2006; Baribeau et al., 2015), but no effect was reported in a more recent study (Warnell and Redcay, 2019).

Concerning the effect of age on RMET–C, results were inconsistent as well: some studies found a significant, positive correlation (Chapman et al., 2006; Baribeau et al., 2015; Durdiaková et al., 2015; Misailidi, 2018; Warnell and Redcay, 2019), whereas others did not (Sharp, 2008; Peterson et al., 2015; Hayward and Homer, 2017; Stevens et al., 2017).

To our knowledge, no previous study focused on the family's influence on children's RMET performance by investigating the effect of parents' education or the presence of older siblings, although parental level of education and the presence of older siblings were extensively taken into account when investigating their influences on children's ToM development, which resulted in mixed results (for a review, Hughes and Devine, 2017).

Concerning the intergenerational transmission of affective ToM, to our knowledge, only one study (Lecciso et al., 2013) has been conducted administering RMET both to mothers and children in a small sample of deaf and hearing children. However, they computed and reported a composite ToM score calculated from different ToM tasks, including RMET. Findings from this study showed that the maternal composite ToM index predicted the same ToM index in deaf children, but not in hearing children.

Two other studies investigated parental influences on children's ToM using RMET. Sabbagh and Seamans (2008) focused on intergenerational transmission of theory of mind skills in a typically developing population and found that parental performance on RMET correlated with the children's performance on a scaled battery of theory of mind tasks (not including RMET) in a sample of 46 children aged 3 and their parents (43 mothers and 3 fathers). Ragsdale and Foley (2011) studied maternal and paternal influences on RMET scores in an adult sample using correlations between pairs of full, maternal and paternal siblings and concluded that there was a maternal influence on RMET performance, although it remained unclear how much of this influence was genetic and how much was environmental.

Given the absence of previous studies on the family influence on children's RMET performance, and because studying the family's influence on children mentalization could have important implications in developing prevention and treatment interventions, this study aims to offer a novel contribution by investigating the effect of parents' education, the presence of older siblings, and of parental performance on RMET.

Because of the inconsistency of the findings from previous studies, the present study was exploratory regarding the effect of sex, age, and intelligence on RMET children's performance. Concerning intelligence, we decided to specifically investigate the effect of verbal ability and the effect of fluid intelligence using two measures designed to assess receptive vocabulary and abstract reasoning through perceptual stimuli.

Regarding the investigation of the relationship between parents' and children's performance on RMET, our study was also exploratory because, to our knowledge, this is the first study aimed at investigating the intergenerational transmission of affective ToM by assessing RMET performance both in children and in their parents.

## Materials and Methods

### Participants

One hundred-twelve mothers (*M* age= 40.77 years ± 4.43; *M* education = 12.53 years ± 3.08), 42 fathers (*M* age = 43.73 years ± 5.19; *M* education = 11.78 years ± 3.49), and their children (55 males and 57 females, aged 77–139 months (*M* = 108.02±17.13) agreed to participate in the study.

Participants were from intact, mostly working class families. Regarding education, only 11 mothers (9.8%) and 11 fathers (9.8%) had obtained a university degree, while 42 mothers (37.5%) and 55 fathers (49.1%) had received an education below the high school level. In this sample, the level of education was lower than the Italian average.

Participation was voluntary and no fee or other incentive was provided for taking part in the study.

None of the participants suffered from psychiatric or neurological illness or severe sensory impairment, none of the children had special educational needs, as reported by the family pediatricians.

### Measures

#### Affective ToM

The RMET was administered to mothers and fathers to assess affective ToM. The Italian version (Vellante et al., 2013) showed acceptable internal consistency (Cronbach's alpha 0.605) and good test-retest stability (ICC = 0.833). One point is assigned to each correct answer, 0 for the wrong or not given answer. The sum of the correct answers, ranging from 0 to 36, was the score used in the current study. The control task Gender Recognition test was also administered to mothers and fathers to control the effect of non-mentalistic social intelligence, as suggested by Baron-Cohen et al. (2001a).

The Italian version (Liverta Sempio et al., 2003) of the RMET-C was used to assess affective ToM in the children. As per administration guidelines, during the task participants could ask the examiner questions and look up a glossary available to them if they needed a better understanding of the words in the test. One point is assigned to each correct answer, 0 for the wrong or not given answer. The sum of the correct answers, ranging from 0 to 28, was the score used in the current study.

The control task Gender Recognition test was administered to the children as well.

#### Receptive Language

The standardized Italian version (Stella et al., 2000) of the Peabody Picture Vocabulary Test—Revised (PPVT-R; Dunn and Dunn, 1981) was used to assess children's receptive vocabulary. PPVT-R consists of 180 cards, each of them presenting four drawings. The child was asked to point to the picture corresponding to the word pronounced by the examiner, and the item was scored 1 point or 0 points if it matched the picture or not, respectively. The examiner stops the test when the child gives eight wrong out of eight consecutive answers.

The task does not involve immediate memory or recall component. Raw scores were used and analyses were performed controlling for age.

#### Fluid Intelligence

The standardized Italian version (Belacchi et al., 2008) of the Raven's Progressive Matrices (CPM; Raven et al., 1998) was used to assess fluid intelligence. It is a non-verbal test of analytic reasoning designed for children aged five to eleven, consisting of 36 items over three sets (A, Ab, B), each including 12 items with increasing difficulty. All items have a missing segment with six possible choices for completion. Children were asked to select the one fitting the drawing best. Raw scores were used and analyses were performed controlling for age.

#### Procedure

The study was presented to three family pediatricians who agreed to collaborate in the research by asking parents and their children (only those not suffering from psychiatric or neurological illness or severe sensory impairment, or having special educational needs) for consent to be contacted by researchers. 70% of parents agreed to be contacted, after which a researcher called them to schedule a meeting. All the families agreed to allow their children to participate. All the mothers agreed to participate as well, while only 42 fathers were willing to be administered RMET. All the children gave their assent. During the first meeting with parents and children, the study was further illustrated, then the RMET was administered to the parents and two subsequent appointments were scheduled to meet the child and to administer in counterbalanced order the RMET-C, the Raven Colored Progressive Matrices, and the PPVT-R. All the participants were met individually at the family pediatricians' office. As per administration guidelines, no time limit was given to complete the tasks, and a break of about 10 min between one task and the next was offered to the children. Each of the two sessions with the children lasted no more than 45 min, and on average, 7–10 days went by between appointments.

Ethical approval for this study was not required in accordance with local legislation and national guidelines. Written informed consent to participate in this study was provided by the participants' legal guardian/next of kin.

## Results

Preliminary analyses of the data indicated that the study variables, except paternal education and scores on Gender Recognition task, were normally distributed with skewness and kurtosis values falling within the accepted range of ± 2 (George and Mallery, 2010), thus appropriate for parametric statistical tests. Descriptive statistics are reported in Table 1.

**Table 1:** Descriptive statistics: Age, Reading the Mind in the Eyes Test, Gender Recognition task, Education, 4 Peabody Picture Vocabulary Test, Raven Progressive Matrices.

	**Mothers** **(*****N*** **=** **112)**		**Fathers (*****N*** **=** **42)**		**Children (*****N*** **=** **112)**	
**Variable**	**M**	**SD**	**M**	**SD**	**M**	**SD**
Age	40.77	4.43	43.73	5.19	108	17.43
RMET	23.82	4.03	22.67	3.67	17.81	3.85
GR task	34.92	1.93	34.48	1.66	26.29	1.88
Educ.	12.53	3.08	11.78	3.49		
PPVT-s					103.13	11.88
PPVT-r					121.71	20.88
CPM-p					61.20	25.55
CPM-r					26.96	4.96

*M, mean; SD, standard deviation; RMET, global score on The Reading the Mind in the Eyes Test; GR task: score on Gender Recognition task; Educ., Years of education; PPVT-s, standard score on Peabody Picture Vocabulary Test; PPVT-r, raw score on Peabody Picture Vocabulary Test; CMP-p, percentile scores on Raven Progressive Matrices; CMP-r, raw scores on Raven Progressive Matrices*.

Only 54 mothers, 12 fathers, and 34 children performed at ceiling on the Gender Recognition test, however no significant association was found between RMET and control task for mothers (*rho* = 0.045, *p* = 0.645), fathers (*rho* = 0.214, *p* = 0.174), or children (*rho* = 0.132; *p* = 0.166). As shown in Table 1, mean values on control task were near to ceiling.

RMET-C internal consistency, as measured by Cronbach's alpha, was 0.593.

A *t*-test was performed to analyze the effect of sex on the variables of interest. No significant effect of sex was found on children's age, scores on RMET-C, PPVT-R, or CPM (*ps* ranging from 0.178 to 0.959).

Since a significant correlation (*r* = 0.391; *p* < 0.0001) was found between age and children's performance on RMET, the effect of sex was investigated again by performing an analysis of covariance with age as the covariate. This analysis confirmed that there was no effect of sex on children's affective ToM (*F* = 0.934, *p* = 0.336).

Partial correlation analysis, controlling for age, was used to investigate the association between RMET-C, PPVT-R, and CPM scores. Results yielded significant correlations between children's performance on RMET-C, PVVT-R (*r* = 0.258, *p* = 0.006), and CPM scores (*r* = 408, *p* < 0.0001). Results are shown in Table 2.

**Table 2:** Partial correlations between RMET-C, CPM, and PPVT scores, controlling for children's age.

	**CPM**	**PPVT**
RMET-C	0.408^***^	0.258^**^
CPM		0.377^***^

CPM, raw scores on Progressive Matrices; PPVT, raw score on Peabody Picture Vocabulary Test; RMET-C, global score on The Reading the Mind in the Eyes Test –Child version.

** p < 0.01;

*** *p < 0.001*.

To explore the extent to which PPVT-R and CPM scores predict RMET-C performance in children, a multiple regression analysis was carried out using children's PPVT-R, CPM scores and age as predictors of the children's RMET-C performance. The final model, shown in Table 3, accounts for approximately 30% of the variance in children's RMET-C score. Specifically, only the CPM score predicted children's RMET-C score (*t* = 3.843, *p* < 0.0001).

**Table 3:** Multiple regression analyses for predicting RMET-C performance.

	**Children's RMET-C score** ***F***_****(**3**, 108)****_ **=15.784;** ***R***^****2****^ **=0.305;** ***p*** **<** **0.0001**				
	***B***	***SE***	**β**	***t***	***p***
**CPM**	**0.291**	**0.076**	**0.387**	**3.843**	**< -0001**
PPVT	0.026	0.020	0.143	1.294	0.198
Age	0.023	0.023	0.106	1.004	0.318

*final model in bold; CMP, raw score on Progressive Matrices; PPVT, raw score on Peabody Picture Vocabulary Test*.

With regard to the effect of the family's influence on affective ToM, partialled correlation analysis, controlling for children's age, was conducted to investigate the association of the children's affective ToM with parents' education and parents' performance on RMET scores. Results yielded no significant correlation, as shown in Table 4.

**Table 4:** Partial correlations between RMET-C, mother's education, father's education, mother's performance on RMET, and father's performance on RMET, controlling for children's age.

	**ME**	**FE**	**M-RMET**	**F-RMET**
RMET-C	0.173	0.140	−0.017	0.140

*ME, mothers' education; FE, fathers' education; M-RMET, global score on the Reading the Mind in the Eyes Test –Adult version reported by mothers; F-RMET, global score on the Reading the Mind in the Eyes Test –Adult version reported by fathers; RMET-C, global score on the Reading the Mind in the Eyes Test –Child version*.

Finally, the effect of having older siblings on affective ToM was investigated using an analysis of covariance with age as the covariate. Forty-six of 112 children had older siblings. No significant effect with regard to older siblings was found (*F* = 0.200, *p* = 0.655).

## Discussion

Preliminary results found that the data produced robust variability in distribution, thus supporting the notion that RMET is not susceptible to the ceiling effect in middle childhood (Baron-Cohen et al., 2001b). Internal consistency for RMET-C was less than acceptable (Devellis, 2012), replicating findings from the Italian validation study of the adult version of RMET (Vellante et al., 2013), and from other studies on the psychometric properties of the children's version (Müller and Gmünder, 2014; Hayward and Homer, 2017), thus raising further questions regarding its unidimensionality. Positive and negative affect subscales were previously hypothesized (for a review, Hudson et al., 2020).

In line with some previous studies (e.g., Chapman et al., 2006; Misailidi, 2018), a significant effect of age was found on children's performance. No effect of sex was found, even when controlling for age, thus replicating Warnell and Redcay (2019) findings in middle childhood.

Significant positive correlations were found between RMET-C performance, fluid intelligence, and receptive language. A significant association between fluid intelligence and RMET-C performance had also previously been found by two studies (Ibanez et al., 2013; Levy and Milgram, 2016). To our knowledge, only two studies on school-aged children (Lecciso et al., 2013; Peterson et al., 2015) used PPVT-R to investigate the association between receptive language and RMET performance. Lecciso et al. (2013) found that receptive language predicted RMET performance, whereas Peterson et al. (2015) did not find any significant associations. In our study, a regression analysis using CPM, PPVT-R, and age as predictors of the children's RMET-C performance revealed that fluid intelligence was the only predictor, and that the model accounts for approximately 30% of the variance. It is noteworthy to point out that the effect of fluid intelligence was observed above and beyond the effect of age. Findings from the current study show that the effect of fluid intelligence on RMET performance, previously reported in a sample of secondary school students (Ibanez et al., 2013) and in two non-clinical adult samples (Bates and Gupta, 2017; Meinhardt-Injac et al., 2020), is also substantial in middle childhood. The effect of fluid intelligence on RMET performance may be related to the fact that RMET involves facial processing, which is also associated with fluid intelligence (Wilhelm et al., 2010), and that both fluid intelligence and social cognition engage the frontal lobe (Roca et al., 2010).

In the current study no family influence was found on children's RMET performance: neither parental education nor the presence of older siblings had an effect on children's scores on RMET. Interestingly, no correlation has ever been found between parents' and children's RMET performance either. A vast array of studies showed that maternal mentalization had a significant effect on children's mentalizing abilities (e.g., Meins et al., 2002, 2003; Ensink et al., 2015; Rosso et al., 2015; Scopesi et al., 2015; Rosso and Airaldi, 2016), and the only previous study investigating the association between mothers' and children's RMET performance (Lecciso et al., 2013) reported a significant correlation in a sample of hearing mothers and deaf children, whereas the association was not found in the hearing dyads. However, unlike our study, Lecciso et al. used a composite ToM index combining RMET and Recognition of Faux Pas (FPT-C; Baron-Cohen et al., 1999), thus findings are not fully comparable. The absence of association between parents' and children's RMET performance observed in our study raises further questions about the diagnostic meaning of the RMET scores. Fonagy and Bateman (2019) reported that both high and low scores on RMET might suggest mentalizing deficits, thereby signaling, respectively, hypermentalizing and hypomentalizing. In fact, a number of studies (e.g., Dinsdale and Crespi, 2013) showed that individuals suffering from Borderline Personality Disorder (BPD) outperformed non-clinical individuals on RMET because of their increased proneness to focus on external features that, in the absence of genuine reflective mentalizing, makes them highly vulnerable in social contexts, generating high interpersonal hypersensitivity. In line with Fonagy and Bateman (2019), it could be argued that the absence of association between parents' and children's RMET performance emerging from our study might be attributable to a non-univocal interpretation of RMET scores, therefore, low scores, like high ones might indicate mentalizing deficits.

On the whole, results from the current study offer some support to the hypothesis proposed by Mary et al. (2016) that RMET-C is not a “pure” ToM task. Specifically, findings from the current study highlight the effect of fluid intelligence on RMET performance, an effect that should be taken into account when RMET is used both in research and in the clinical setting.

## Data Availability Statement

The raw data supporting the conclusions of this article will be made available by the authors, without undue reservation.

## Ethics Statement

Ethical review and approval was not required for the study on human participants in accordance with the local legislation and institutional requirements. Written informed consent to participate in this study was provided by the participants' legal guardian/next of kin.

## Author Contributions

ARo designed the study, performed the statistical analyses, and wrote the article. ARi contributed to the search for references, coordinated data collection and scoring, and contributed to the final version. Both authors contributed to the article and approved the submitted version.

## Conflict of Interest

The authors declare that the research was conducted in the absence of any commercial or financial relationships that could be construed as a potential conflict of interest.
